# Photodynamic therapy with phthalocyanine sensitisation: quantitative studies in a transplantable rat fibrosarcoma.

**DOI:** 10.1038/bjc.1987.78

**Published:** 1987-04

**Authors:** C. J. Tralau, A. J. MacRobert, P. D. Coleridge-Smith, H. Barr, S. G. Bown

## Abstract

**Images:**


Br. J. Cancer (1987), 55, 389-395 © The Macmillan Press Ltd., 1987

### Photodynamic therapy with phthalocyanine sensitisation: quantitative studies in a transplantable rat fibrosarcoma

C.J. Tralau[1], A.J. MacRobert[2], P.D. Coleridge-Smith[1], H. Barr[1] & S.G. Bown[1]

[1]*National Medical Laser Centre, Faculty of Clinical Sciences, University College, and the* [2]*Royal Institution, London, UK.*

**Summary** Photodynamic therapy (PDT) is a promising approach to the local destruction of malignant tumours, but little work has been done to determine which factors control the extent of tissue necrosis produced. Using a new photosensitiser, a sulphonated aluminium phthalocyanine (AlSPc) and light from an argon ion pumped dye laser at 675 nm, we quantified the effects of interstitial PDT in a transplantable fibrosarcoma in rats. At 100 mW laser power, thermal effects were comparable to those of PDT, so subsequent studies were carried out at 50 mW, where thermal effects were minimal. The depth of PDT necrosis increased with the logarithm of the applied energy. Tissue concentration of AlSPc was measured by alkali extraction and at all times after sensitisation, correlated well with the necrosis produced with a given light dose. Peak tumour concentration of AlSPc occurred 24–48 h after sensitisation compared with a peak at 3 h in muscle. The peak ratio tumour:muscle was 2:1 at 24 h. Apart from a different time interval to reach the peak sensitiser concentration, the extent of tumour damage varied with the light and sensitiser parameters in a similar way to that found in normal liver, although the optical penetration depth was greater in the tumour (2.5 mm *vs.* 1.8 mm). At doses of AlSPc below 1 mg $kg^{-1}$ the diameter of necrosis increased with the logarithm of the dose of sensitiser, and doubling the dose from 0.25 to 0.5 mg $kg^{-1}$ increased the depth of necrosis by 50%. However, at higher doses, the changes were smaller and increasing the dose from 2.5 to 5 mg $kg^{-1}$ only increased the necrosis by 10% for the same light dose. In all dose ranges, a given percentage increase in the tissue concentration of AlSPc gave a much smaller percentage increase in the extent of necrosis for the same light dose, suggesting that selectivity of necrosis between tumour and normal tissue is likely to be much less than the selectivity of retention of the photosensitiser. From these results, the extent of PDT necrosis in this fibrosarcoma is as predictable as it is in normal liver if the light dose, tissue concentration of AlSPc and optical penetration depth of the tissue are known. Further studies are now required on different tumour models to establish how tumours respond compared with adjacent normal tissue when the tumour is growing in its organ of origin rather than the non-physiological situation using a transplantable tumour as in this study.

Photodynamic therapy (PDT) is a method of treating tumours by the combined use of systemically administered photosensitisers and the local application of light. It has been suggested that porphyrins, in particular haematoporphyrin derivative (HpD), accumulate selectively in malignant tissue, so causing these tumours to fluoresce (Gregorie *et al.*, 1968). When irradiated with an appropriate wavelength of light these photosensitisers are excited to the triplet state which is then capable of reacting directly with tissue components or undergoing interaction with molecular oxygen in order to produce cytotoxic species such as singlet oxygen and free radicals (Weishaupt *et al.*, 1976). Most of the work reported to date has used HpD (Lipson *et al.*, 1961) as the sensitiser, but HpD is far from ideal. It is a mixture of porphyrins, it is difficult to keep its composition the same in different batches, tumour selectivity is poor and it only has a weak absorption peak in the red area of the spectrum where tissue penetration is high. For these reasons we decided to look closer at a new group of photosensitisers, the phthalocyanines. In a recent review of phthalocyanines, Spikes (1986) suggests that these compounds could be valuable sensitisers for PDT of tumours. A number of studies have shown that various phthalocyanines (PC) will photosensitise the killing of mammalian cell lines in culture. Ben-Hur and Rosenthal (1985, 1986) have reported studies on Chinese hamster fibroblasts V-79, using chloraluminium PC(CAPC), tetrosulfo PC (TSPC) and its metal chelates. They reported that the metal free dye (TSPC) was 4 times more effective than haematoporphyrin (HP) and chelation with metal ions generally resulted in diminished photosensitising capacity except with cerium. The cerium derivative was 5 times more effective than the metal free PCs. Chan *et al.* (1986) used aluminium chloro sulphonated PC (AlSPc) with two murine cell lines N1H/3T3 ('normal fibroblast line') and UV-2237 (a 'transformed' fibrosarcoma line) to study cell killing and uptake of the AlSPc. Both lines behaved similarly and those sensitised with AlSPc were killed more rapidly by light than those treated with a similar dose of HpD.

A number of phthalocyanines have been shown to be selectively retained in malignant tissues. Frigerio (1962) showed selective uptake of water soluble SPC in inplanted brain tumours of mice and reported a ratio tumour:normal brain of 50:1. Rousseau *et al.* (1983, 1985) and Van Lier & Rousseau (1984) used technetium ($^{99m}$Tc) and gallium ($^{67}$Ga) radiolabelled TSPC in the 13762 mammary adenocarcinoma of Fischer 344/CRBL rats. Tc-TSPC achieved ratios of 3.8:1 tumour:muscle at 24 h post-sensitisation and Ga-TSPC achieved a ratio of 15:1 tumour:muscle at 6 h post-sensitisation. Straight and Spikes (reported in Spikes, 1986) found selective retention of zinc SPc in S180 tumours of Swiss Webster mice although the tumour:normal tissue ratio is not stated nor is the time at which maximum selectivity is found. We have shown selective retention of AlSPc in dimethylhydrazine induced colon tumours in Wistar rats, a ratio of 2:1 tumour:normal colon being found at 24–48 h (Tralau *et al.*, 1986) and we have also shown a 2:1 ratio, tumour:muscle in a fibrosarcoma of rats at 48 h as described later in this paper. Little data have yet been reported on *in vivo* phthalocyanine phototherapy. Spikes (1986) reports a personal communication from Van Lier indicating that metal free, TSPC, AlTSPC, cerium TSPc and HpD (Photofrin II) have been compared as photosensitisers of a mouse (BALB/c) mammary tumour (EMT-6) and that AlTSPC is as effective and cerium TSPc more effective than HpD. In the same paper Spikes also reports studies of ZnSPc as a sensitiser of implanted S180 tumours in mice. They have shown that 220 J/CM2 of 650 nm laser light decreases the

Correspondence: C.J. Tralau, Department of Surgery, The Rayne Institute, University College London, 5 University Street, London WC1, UK.
Received 29 September 1986; and in revised form, 24 November 1986.

tumour volume by 50% whereas a dose of 360 J/CM2 of 630 nm light is required to produce the same effects with HpD. Unfortunately the doses of sensitisers used are not reported nor is the laser power and for meaningful results the doses must be comparable, and hyperthermic effects eliminated. There have been few quantitative studies done on photodynamic therapy. In 1984 we reported preliminary quantitative studies in liver, muscle and fibrosarcoma of CBH rats (Coleridge-Smith *et al.*, 1984). At 48 h after sensitisation with $20\,mg\,kg^{-1}$ HpD given i.v., all 3 tissues studied had a similar area of necrosis for similar light doses.

Pimstone *et al.* (1982) showed that with haematoporphyrin sensitisation the depth of necrosis in normal liver increased with the natural logarithm of the applied energy and we showed similar results in normal liver with both HpD and AlSPc sensitisation (Bown *et al.*, 1986). Analysis of the parameters involved showed that the factors which effected the extent of PDT necrosis were: (1) Dose of sensitiser; (2) Time from sensitisation to phototherapy; (3) Power and exposure time of light source; (4) Circulation through the tissue during light exposure.

AlSPc was chosen for our studies because it is easy to synthesise, chemically stable and has a strong absorption peak (Q band) in the red part of the spectrum at 675 nm in aqueous solution. It has a good fluorescence quantum yield (0.6) in aqueous solution needed for fluorescence localisation of tumours; and has a good triplet quantum yield (0.4) with a long lived triplet state ($510\pm50\,\mu$SEC, pH 7.4) which is capable of undergoing energy transfer to produce cytotoxic species (McCubbin, 1985). The purpose of this paper is to look at which parameters effect the extent of PDT induced necrosis in malignant tissue (the transplantable fibrosarcoma) in rats and to compare the results with those obtained previously in normal liver. Such a study has not been reported before.

### Materials and methods

### *Tumour model*

The tumour model used throughout was a transplantable fibrosarcoma (HSN/TC/7) of CBH rats. Solid tumour tissue (1 g) was removed immediately after killing the donor animal and mechanically disaggregated in a laboratory mixer emulsifier (Silverson Machines Ltd.) on medium speed in 10 ml of 0.9% saline. This solution (0.1 ml) was injected s.c. into both flanks of rats anaesthetised with i.m. hypnorm $0.5\,ml\,kg^{-1}$ (fentanyl and fluanisone). Rats were given food and water *ad libitum*. The tumour took 10–12 days to grow to 6–10 mm in diameter. This size was used for quantitative studies as no spontaneous necrosis was seen. At larger diameters, spontaneous necrosis was seen frequently making quantitative studies difficult, although it was usually possible to tell the difference between spontaneous and PDT induced necrosis on histological grounds.

### *Photosensitiser*

Aluminium chloro sulphonated phthalocyanine (AlSPc) as supplied by Ciba Geigy was used. This has an average of 3 sulphonic acid groups per molecule (McCubbin, 1985). This was dissolved in 0.9% saline and administered by i.v. injection into a tail vein. Sensitised animals were kept in subdued lighting.

### *Pharmacodynamics of AlSPc*

The concentration of AlSPc was measured in tumour, adjacent muscle and skin and in plasma at various times from a few minutes to 120 h post sensitisation with $5\,mg\,kg^{-1}$ AlSPc and in tumour after sensitisation with various doses of AlSPc ($0.2$–$25\,mg\,kg^{-1}$). Rats were killed by cervical dislocation and $\sim0.5$ g each of tumour, underlying muscle and overlying skin were removed and kept frozen at $-20$°C until extraction. Tissue samples were weighed, finely chopped and homogenised in 7 ml of 0.1 M NaOH for 2 min. The homogenate was centrifuged at 12,000 rpm for 5 min at 4°C, the clear supernatant separated, and the fluorescence read at 675 nm on a spectrofluorimeter (Perkin Elmer LS–5 Luminescence Spectrophotometer) with excitation at 610 nm. We have previously shown that this is an efficient extraction procedure (Bown *et al.*, 1986). The effect of quenching of fluorescence of solutions of known concentrations of AlSPc in 0.1 M NaOH was compared with that from the same concentrations of AlSPc when present in the supernatant from the extraction procedure on unsensitised tissues. The percentage quenching found remained the same throughout the standard curve and the mean quenching for each tissue was 18% (tumour), 19% (skin) and 16% (muscle). In order to correct for this all results from sensitised tissues were calibrated against a standard curve of known concentrations of AlSPc in tissue supernatant (unsensitised) and expressed in micrograms of AlSPc extracted per gram of tissue.

### *Phototherapy*

The light source used was an argon ion pumped dye laser (Aurora-Cooper Lasersonics). The dye used was DCM (4-dicyanomethylene-2-methyl-6-(p-dimethylaminostyryl)-4H pyran in ethylene glycol and propylene carbonate) and the laser was tuned to emit light at 675 nm. The light was delivered via a 0.2 mm diameter quartz fibre with a plastic coating left on to within 1 mm of the fibre end to prevent emission of light from any region other than the last 1 mm. The fibre was cleaved as often as necessary to obtain a clean circular light beam and the power checked in a power meter (photon control) prior to each treatment. Animals were anaesthetised as for sensitisation, the skin of the area surrounding the flank tumours was shaved and the tumour exposed by a single incision of the overlying skin. The size of the tumours was measured in three dimensions, using calipers, although most were roughly spherical. The 'capsule' of the tumour was pierced with a needle point and the fibre inserted into the middle of the tumour mass. Control experiments using 50 and 100 mW were done in unsensitised animals to establish the power at which purely thermal effects could be seen. Fifty mW was chosen as the power which limited thermal effects to a zone $<2$ mm in diameter around the fibre tip and which did not cause charring. The laser was therefore set prior to fibre insertion to give a power output of 50 mW at the fibre tip for the quantitative studies in sensitised animals. After exposure the fibre was removed, the skin incision sutured and the animal allowed to recover from the anaesthetic. The animals were re-anaesthetised and given an i.v. injection of Evans blue a few minutes before death (1 ml 0.5% Evans in 0.9% saline) to facilitate subsequent identification of necrotic areas. Animals were killed by cervical dislocation at various times from treatment (0.1–96 h) and tumour tissue removed immediately, fixed in 10% formalin, sectioned at 1 mm intervals and the maximum and minimum width of macroscopic necrosis (white area) was measured in each slice. The highest mean value (average of maximum and minimum) was recorded. Representative sections were sent for histological examination. Microscopically measured areas correlated well with those measured macroscopically. At the power level where thermal effects were negligible, experiments were carried out to investigate the effects of the following parameters on the extent of necrosis produced:

(1) Laser energy (1 to 200 J), for one laser power (50 mW), one dose of sensitiser ($5\,mg\,kg^{-1}$), and at one time from sensitisation to phototherapy (3 h).

(2) Dose of sensitiser ($0.2\,mg\,kg^{-1}$ to $25\,mg\,kg^{-1}$) for one laser power (50 mW) one energy (50 J) and one time from sensitisation to phototherapy (3 h).

(3) Time from sensitisation to phototherapy (6 min to

72 h) at one dose of AlSPc (5 mg $kg^{-1}$) and one laser power (50 mW) and one energy (50 J).

### Results

The AlSPc pharmacodynamic results given are those for extractable fluorescence of phthalocyanine when excited at 610 nm and emission read at 675 nm. Although the AlSPc solution consists of only aluminium chloro sulphonated phthalocyanines, it contains varying numbers of sulphonic acid groups ($-SO_3^-$) per molecule. It is understood that the number of $-SO_3^-$ groups will effect the distribution within the animal so the results are given as the best approximation for the combination of phthalocyanine species present in the tissues.

The quantity of AlSPc extracted from tumour, overlying skin and underlying muscle as a function of time from sensitisation is summarised in Figure 1 and detailed in Table I. The concentration of AlSPc peaks in tumour tissue at 24–48 h post-sensitisation whereas muscle peaks after 3 h. The total quantities are higher in skin than tumour and muscle throughout the time period studied. A similar pattern was found for skin and muscle in non-tumour animals.

**Figure 1** Concentration of extractable AlSPc in tumour (●), muscle (○), skin (□) and plasma (▲) following i.v. administration of 5 mg $kg^{-1}$ AlSPc.

The maximum tumour:muscle ratio is achieved at 24–48 h and is 2:1. The plasma data is shown for comparison. An example of macroscopic tumour necrosis brought out by the Evans blue stain is shown in Figure 2. The relationship between mean width of necrosis and applied energy (= power × time) at a power of 100 mW and 50 mW is shown in Figures 3 and 4 respectively, for an AlSPc dose of 5 mg $kg^{-1}$ and time from sensitisation to treatment of 3 h. It can be seen that the control value (unsensitised tumour) at a power of 100 mW, and energy 50 J is 4.5 mm mean diameter whereas the control value at 50 mW for the same energy is

**Figure 2** Section of treated tumour following i.v. administration of Evans blue 10 min before death and subsequent fixation in formalin. The area of PDT necrosis (N) is distinguishable from viable tumour (T) (×8).

**Figure 3** Mean diameter of laser induced necrosis in tumour as a function of applied energy for exposure at a laser power of 100 mW, 3 h after sensitisation with 5 mg $kg^{-1}$ AlSPc (●); controls in unsensitised tumours are also shown (○). Each point represents the mean (±s.d.) of at least 5 animals.

**Table I** Concentration of extractable AlSPc in skin, tumour, muscle and plasma, measured by alkali extraction after i.v. administration of 5 mg $kg^{-1}$ (N) $\bar{x}$±s.d.

| *Time from administration (h)* | *AlSPc* Tumour | *Muscle μg g⁻¹* | *Skin* | *Plasma μg ml⁻¹* |
|---|---|---|---|---|
| 0.1 | — | — | — | (3) 2.7±0.5 |
| 1 | (4) 2.9±0.3 | (4) 2.3±0.8 | (4) 5.7±1.3 | (3) 1.2±0.5 |
| 3 | (3) 3.0±0.3 | (3) 3.2±0.3 | (3) 4.9±2.3 | (3) 0.5±0.7 |
| 24 | (3) 3.1±1.0 | (4) 2.3±1.7 | (4) 5.4±2.0 | (3) 0.3 |
| 48 | (3) 3.9±0.5 | (3) 1.4±0.6 | (2) 4.4±1.5 | (3) 0.0 |
| 72 | (4) 2.8±0.3 | (4) 1.4±0.5 | (4) 4.8±1.6 | (3) 0.0 |
| 96 | (4) 1.4±0.2 | (4) 2.3±0.5 | (4) 4.6±0.6 | (3) 0.0 |
| 120 | (4) 2.1±0.8 | (4) 2.1±1.8 | (4) 4.2±1.4 | (3) 0.0 |

Figure 4 Mean diameter of laser induced necrosis in tumour as a function of applied energy for exposure at a laser power of 50 mW, 3 h after sensitisation with 5 mg kg$^{-1}$ AlSPc (●); controls in unsensitised tumours are also shown (○). Each point represents the mean (±s.d.) of at least 5 animals.

Figure 5 Mean diameter of laser induced necrosis in tumour (50 mW, 50 J) as a function of the time from sensitisation (AlSPc 5 mg kg$^{-1}$) to light exposure. Each point represents the mean (±s.d.) from at least 3 animals.

only 2.5 mm. Thermal effects are obviously involved at 100 mW and as we wished to investigate effects due to PDT alone a power of 50 mW was used for all further work to minimise thermal necrosis. The variation of mean width of necrosis in relation to time from sensitisation to phototherapy is shown in Figure 5. The maximum width of necrosis was seen when phototherapy was carried out 24 h post sensitisation. The effect of varying the dose of AlSPc (0.2 to 25 mg kg$^{-1}$) on mean width of necrosis is shown in Figure 6. The concentration of AlSPc in tumours 3 h after the administration of various doses of AlSPc (0.2 to 25 mg kg$^{-1}$) is shown in Figure 7.

Figure 6 Mean diameter of laser induced necrosis in tumour (50 mW, 50 J, 3 h after sensitisation) as a function of administered dose of AlSPc (●); control value in unsensitised tumour is also shown (○).

Figure 7 Concentration of extractable AlSPc (μg g$^{-1}$ tissue) as a function of dose, 3 h after administration. Each point represents the mean of 2 tumours.

### Discussion

The aim of this paper was to establish whether the parameters controlling the extent of PDT necrosis in malignant tissue are the same as those controlling the damage in normal tissue, that we have reported previously in studies on the liver (Bown *et al.*, 1986). The factors involved can be divided into those related to the light dose and the sensitiser.

### *Light dose*

Using the interstitial method of treatment with the laser fibre inserted into the target tumour, it is inevitable that some thermal effects will be seen in the immediate vicinity of the fibre tip. In liver, at a laser power of 100 mW, thermal damage in unsensitised animals was limited to a zone about 2 mm in diameter, so the greater damage seen in sensitised animals could be attributed to photodynamic effects. However, in the tumour model at the same power, the zone of necrosis increased in diameter with increasing energy even in unsensitised animals (Figure 3) showing the tumour to be more sensitive to hyperthermia than the liver. There are several possible explanations for this. These tumours are known to develop spontaneous central necrosis when over 1 cm in diameter so even when less than this size, as used in these experiments, the centre, where the fibre tip is located during therapy, may be more vulnerable to thermal damage than normal liver. Also, the high blood flow in liver may remove heat from the zone around the fibre tip more effectively, although our previous studies occluding the blood supply to the liver during phototherapy did not show any increase in thermal damage in the liver without blood flow. However, the most important conclusion from these results is that the threshold power for thermal damage varies between different organs. In our tumours, if the power was kept down to 50 mW, thermal damage was limited to a zone of about 2 mm in diameter, so any greater damage could be attributed to photodynamic effects.

With the assumption that a threshold energy dose $W_\tau$ for necrosis by PDT is reached at the edge of the necrotic zone, using diffusion theory (Svassand *et al.*, 1984) $W_\tau$ is related to $r$, the radius of the necrotic zone according to:

$$W_\tau = A(W_o/r)\exp(-(r-d)/d)$$

where $W_o$ is the total energy dose, $d$ is the penetration depth and $A$ is a constant. From our previous analysis (Bown *et al.*, 1986) this expression can be rearranged when $r \approx d$ to give with good approximation

$$2r = d \ln W_o + \text{Constant}$$

From Figure 4, for energies over about 10 J this gives a value of $d = 2.1 \pm 0.3$ mm for the tumour. Alternatively, without making approximations the expression is simply rearranged to give,

$$r = d \ln(W_o/r) + \text{Constant}$$

Note however that errors in the value of $r$ now appear in both sides of the equation. Analysis of the data for $W_o$ and $r$ yields a value of $d = 1.8 \pm 0.3$ mm which is close to that derived with the first method which although approximate has the merit of facilitating interpretation of the energy dose dependence shown in Figure 4.

Thse use of diffusion theory is however only valid for values of $r$ where the light distribution has become isotropic through scattering. In practice, close to the fibre tip, the distribution is not isotropic as the beam is roughly collimated as it emerges from the fibre (Svaasand & Ellingsen, 1983). This would explain the necrosis observed at the relatively low energy of 1 J. This value of $d$, $\approx$2 mm, may also be an underestimate due to significant absorption by the AlSPc, as discussed below. The data shown in Figure 3 for 100 mW are more difficult to interpret since thermal effects cannot be ignored and there is the possibility of charring or coagulation occurring at the fibre tip which would alter and distort the light distribution. Even though the optical penetration of light at 675 nm is greater in tumour than liver, the absolute quantity of AlSPc is less in tumour than liver (1:2.7, 3 h after 5 mg kg$^{-1}$ AlSPc). The size of the necrotic zone depends on both factors and in this case is slightly wider in tumour than it is in liver for the same energy (8.5 mm *vs.* 7.5 mm, for 100 J 3 h after 5 mg kg$^{-1}$ of AlSPc). The extent of necrosis around one treatment site can be increased with higher energies, but as the power cannot be increased without introducing thermal effects, extremely long exposure times would be necessary, and it would be much easier to treat larger volumes by using multiple treatment sites.

There have been several reports that PDT and hyperthermia are synergistic and there is certainly evidence to support this (Henderson *et al.*, 1985), but until the effects of both are more fully understood, we feel it is wise to restrict studies to situations in which only one form of tissue damage is investigated at a time. If the tumour to be treated is very close to the surface, the light can be delivered without the need for a fibre to be in direct contact with tissue, and a larger area can be illuminated from a single light source without the need for multiple fibres. Indeed, this is the approach taken for most experimental and clinical work with PDT. The risk of thermal effects is much less, but is certainly still present and control studies are always necessary to see whether they are occurring or not. Also, it is often difficult to achieve a uniform light intensity across an extended surface. The depth of tissue necrosis, as for interstitial treatment, will depend on the optical penetration depth, the light fluence and the local concentration of sensitiser. Although the geometry is different, the depth of necrosis is likely to be comparable to the radius of necrosis around an interstitial fibre for similar treatment parameters. This means that for easily achieved light levels, the depth of necrosis is likely to be less than 5 mm from the surface.

*Sensitiser*

The pattern of AlSPc accumulation in this fibrosarcoma is that of a gradual increase to a peak at 24–48 h after administration followed by an equally gradual decrease (Figure 1). The concentration of plasma AlSPc is at its highest at 0.1 h after sensitisation: it is therefore unlikely that the tumour peak is due to plasma levels. AlSPc, which is initially bound to plasma proteins (W.S. Chan, Personal Communication) is taken up from the plasma into the tumour tissue possibly by non-specific binding of serum proteins to stromal elements and leaky vasculature (Bugelski *et al.*, 1981). The slow release of AlSPc from tumour tissue may be due to (i) poor lymphatic drainage (Bugelski *et al.*, 1981); (ii) binding to tissue components such as (a) lipoprotein receptors (Jori *et al.*, 1984); or (b) collagen and elastin (El Far & Pimstone, 1985). However, details of the factors controlling the tissue distribution of these photosensitisers are still poorly understood.

The tumour we used is transplantable and fast growing and may behave quite differently from human neoplasms. Nevertheless, studies such as the present one require similar tumours in a large number of animals which is only possible with a tumour model such as the one used. In addition, the peak ratio of AlSPc concentration we found between tumour and muscle (2:1 at 24–48 h post sensitisation) is comparable to our results for an autochthonous tumour (colon cancer induced by dimethylhydrazine in rats) in which the peak ratio between tumour and normal colon was also 2:1 at 24–48 h post sensitisation (Tralau *et al.*, 1986). This compares well with results reported for HpD. In dimethylhydrazine induced colonic tumours of mice the ratio was 1.8:1 at 72 h post i.p. administration of 10 mg kg$^{-1}$ HpD (Agrez *et al.*, 1983) and a similar ratio, 1.8:1 (tumour:muscle), was reported in methylcholanthrene induced mammary carcinoma of mice 24 h post i.p. administration of 10 mg kg$^{-1}$ HpD (Gomer & Dougherty, 1979).

It is reassuring that the concentration of AlSPc measured by extraction at different times after sensitisation correlates well with the extent of the necrosis produced by a given light dose (50 J at 50 mW) at the same times after sensitisation (Figures 1 & 5). The peak concentration was seen 24–48 h after sensitisation, and maximum necrosis with a 24 h interval. We found the same correlation in the studies on normal liver, although in liver the maximum of both extractable concentration of AlSPc and necrosis was spread over a much longer time period.

The total dose of AlSPc influences the extent of PDT damage as shown in Figure 6. At 0.2 mg kg$^{-1}$ the necrosis does not differ significantly from that in unsensitised animals, but at higher doses increases up to the maximum dose tolerated by the animals, which was 25 mg kg$^{-1}$. In liver, the extent of necrosis for a given light dose fell for doses above 5 mg kg$^{-1}$ due to absorption of light by the large amounts of AlSPc in the tissue which reduced the optical penetration depth. This was not seen in the tumours presumably because the dose required to achieve this was above the dose that would kill the animal by a direct toxic effect although at the higher doses of AlSPc the damage does not increase so rapidly with the dose as it does at the lower doses.

For photosensitiser doses which are sufficiently low for the tissue absorption to be dominant over that of the photosensitiser, the light energy required to cause necrosis is

$$W_o = K\varphi tC \quad (1)$$

where $C$ is the photosensitiser concentration ($\mu g\,g^{-1}$) $\varphi$ is the energy flux density or space irradiance, $t$ is the irradiation time and $K$ is a constant which depends on several factors including the sensitiser absorption coefficient and singlet oxygen yield (Profio & Doiron, 1981). At the edge of the necrosis zone of radius $r$, the space irradiance is given by:

$$\varphi_r = A(d/r)\exp(-(r-d)/d) \quad (2)$$

where $A$ is a constant and $d$ is the optical penetration depth at the wavelength of the light source (Svaasand *et al.*, 1984).

In the case where the total delivered light dose remains constant substitution of (2) in (1) gives:

$$C = B(r/d)\exp((r-d)/d) \text{ where } B \text{ is a constant.}$$

This assumes that the photosensitiser absorption is small enough to neglect a change in the value of $d$. Thus:

$$\ln C = \ln(r/d) + (r-d)/dt + \ln B$$

When $r \approx d$ this expression may be simplified using the approximation

$$\ln r/d \approx r/d - 1$$

so that

$$\ln C = 2r/d - 2 + \ln B$$

and

$$2r = d\ln C + 2d - d\ln B$$

Thus for the given conditions with a constant total energy delivered from the fibre, the diameter of the necrotic zone will increase approximately with the logarithm of the photosensitiser dose with gradient $d$, the penetration depth. From Figure 6 for doses less than $1\,mg\,kg^{-1}$ the diameter of necrosis increases with the logarithm of the dose with an approximate gradient of 2.5 but at higher doses shows a reduced rate of increase with dose. These lower than expected values found at higher doses can be ascribed to light attenuation by the AlSPc itself which absorbs strongly at 675 nm (the molar extinction coefficient in aqueous solution is $1.7 \times 10^{-5}\,M^{-1}\,cm^{-1}$ (Darwent *et al.*, 1982).

To model the dependence of necrosis on dose when the sensitiser absorption is significant requires measurement of several parameters including the sensitiser absorption coefficient in the tissue, and the tissue absorption and scattering coefficients. The possibility of sensitiser photodecomposition should also be taken into account. These measurements have not been made, but several conclusions of practical importance can be made on the basis of the present analysis and the data in Figure 6. Firstly, it is clear that at the higher doses of AlSPc, absorption of light by the AlSPc itself does become comparable with the tissue absorption to explain why the graph flattens at the higher doses, instead of remaining linear. This could also explain why the value of $d$ from Figure 6 (2.5 mm) is greater than that obtained from Figure 4 (2.1 mm) as in the latter case, the dose of AlSPc used was $5\,mg\,kg^{-1}$, which is high enough for absorption by the AlSPc to be significant compared with that by the tumour itself, and so reduce the overall penetration depth. Thus, when the AlSPc dose increases from 0.25 to $0.5\,mg\,kg^{-1}$, the width of necrosis increases from 3.3 to 5.0 mm (50%). However, when the dose increases from 2.5 to $5\,mg\,kg^{-1}$, the necrosis only rises from 6.0 to 6.6 mm (10%). It can be seen from Figure 7 that the amount of extractable AlSPc 3 h after administration was approximately proportional to the administered dose, at least for doses below $5\,mg\,kg^{-1}$. Therefore Figure 6 also represents the relationship between the tissue concentration of AlSPc at the time of light exposure and the resultant necrosis. For maximum selectivity of tumour damage relative to adjacent normal tissue one wants the maximum difference in necrosis for a given difference in tissue concentration of sensitiser. It is clear that this occurs on the linear portion of Figure 6 at the low doses of AlSPc where absorption by AlSPc is small compared with absorption by the tumour itself. No comparable studies have been performed with HpD, but the one other study that looked at the effect of varying the dose of sensitiser (Pimstone *et al.*, 1982), showed that the depth of necrosis in normal liver also increased with the logarithm of the dose of sensitiser (haematoporphyrin, in that case). However, the absorption coefficient of HpD at 630 nm is much lower than that of AlSPc at 675 nm, so absorption by HpD may not be comparable to that of the tissue itself until much higher tissue levels are reached. This means that tumour selectivity with HpD will not depend so critically on the dose of HpD used as is the case for AlSPc. However, high doses of HpD cause major problems of cutaneous photosensitivity, and so are best avoided if possible. In normal liver, the light energy required to produce a zone of necrosis 4 mm wide is 10 times greater 3 h after sensitisation with $5\,mg\,kg^{-1}$ HpD than it is 3 h after the same dose of AlSPc.

El Far and Pimstone (1985) reported that a number of tumour localising porphyrins (Uroporphyrin, HpD, Photofrin II) have a high affinity for collagen and elastin and AlSPc may bind to these components of skin. A major problem of PDT using HpD is skin photosensitivity and as there are high levels of AlSPc in skin one might expect skin photosensitivity to be a major problem with this sensitiser as well. When mice were given [$^{14}$C] and [$^{3}$H]-HpD at a dose of $10\,mg\,kg^{-1}$ i.p., lower absolute levels were found in the skin (Gomer & Dougherty, 1979) than we found with AlSPc ($5\,mg\,kg^{-1}$, i.v.). However, during our earlier experiments it was noted empirically that animals sensitised with $5\,mg\,kg^{-1}$ HpD had a greater cutaneous photosensitivity reaction in room light than those sensitised with the same dose of AlSPc (Bown *et al.*, 1986). Also, Chan *et al.* (1986) reported that cells incubated in AlSPc were killed when irradiated at 675 nm but were less sensitive to white light than cells treated with equal concentrations of HpD. Thus, the high levels of AlSPc found in skin may not be as much of a problem as would be expected, but more detailed studies of the skin response to light in sensitised animals are required to clarify this.

In conclusion, this study has shown that the extent of PDT necrosis varies with the dose of sensitiser (AlSPc), and the laser power and exposure time in a similar way in normal liver and in a transplantable malignant tumour. It has also shown that there is good correlation of tissue damage with the tissue concentration of sensitisor at the time of phototherapy (as measured by alkali extraction). Thus it is possible to predict the extent of necrosis that will be produced with given treatment parameters in both normal and neoplastic tissue which is a significant step forward. AlSPc distribution studies have shown a peak ratio of 2:1 between fibrosarcoma and muscle and between colon cancer and normal colon which is comparable selectivity to that shown with HpD. The difference in tissue damage for a change in tissue concentration of sensitisor by a factor of 2 is greater when the absolute values of tissue levels of AlSPc are lower rather than higher, but even so, is less than 2, 1.5 being the greatest ratio shown in this study.

Selective tumour destruction may be possible by other means. More selective tumour retention of the sensitiser might be achieved by administering the sensitiser in liposomes (Jori *et al.*, 1986) or bonded to monoclonal antibodies (Mew *et al.*, 1983). Also selectivity can be increased by directing the therapeutic light at the tumour and not at the surrounding normal tissue, although to eradicate a tumour locally, the most important area to treat is where that tumour meets the normal tissue. The other possibility is that the normal and tumour areas will respond differently to the same local light fluence and concentration of sensitiser. This is best tested in autochthonous tumours, so the tumour develops in its organ of origin instead of being transplanted, but no such work has yet been reported. Of course, it does not matter if both normal and tumour tissue are destroyed if the tumour is completely ablated, normal tissue regenerates, and adequate structure and function of the treated organ is maintained at all stages of healing. However, for this to be achieved, further detailed studies are required of the healing of relevant normal tissues after PDT damage.

Overall, these results suggest that destruction of malignant tumours based on selective retention of photosensitisers in the neoplastic areas is likely to have less selectivity than has been claimed hitherto. Nevertheless, local non-thermal tumour destruction of predictable extent is possible and the technique of PDT warrants further laboratory investigation, particularly to study the response of small tumours induced in clinically relevant organs such as the colon, bladder, lungs and pancreas.

This work was funded by the Imperial Cancer Research Fund and undertaken in the Department of Surgery, University College London. The project was carried out in close collaboration with Dr W.S. Chan and Dr I.R. Hart at the Imperial Cancer Research Fund Laboratories (Lincolns Inn Fields, London), Mr R. Svensen and Prof D. Phillips at the Royal Institution and Dr T.N. Mills in the Department of Medical Physics at University College Hospital. In addition, Dr A. MacRobert was supported by a training fellowship from the Medical Research Council and Mr H. Barr by a grant from the Wellcome Trust. We should also like to thank the Stanley Thomas Johnson Foundation for a generous grant towards the purchase of the laser.

**References**

AGREZ, M.V., WHAREN, R.E., ANDERSON, R.E. & 5 others (1983). Hematoporphyrin derivative. Quantitative uptake in dimethylhydrazine induced murine colorectal carcinomata. *J. Surg. Oncol.*, **24**, 173.

BEN-HUR, E. & ROSENTHAL, I. (1985). Photosensitised inactivation of Chinese hamster cells by phthalocynines. *Photochem. Photobiol.*, **42**, 129.

BEN-HUR, E. & ROSENTHAL, I. (1986). Action spectrum (600–700 nm) for chloroaluminium phthalocyanine induced phototoxicity in Chinese hamster cells. *Lasers Life Sciences*, **1**, 79.

BOWN, S.G., TRALAU, C.J., COLERIDGE-SMITH, P.D., AKDEMIR, D. & WIEMAN, T.J. (1986). Photodynamic therapy with porphyrin and phthalocyanine sensitisation: Quantitative studies in normal rat liver. *Br. J. Cancer*, **54**, 43.

BUGELSKI, P.J., PORTER, C.W. & DOUGHERTY, T.J. (1981). Autoradiographic distribution of HpD in normal and tumour tissue of the mouse. *Cancer Res.*, **41**, 4606.

CHAN, W.S., SVENSEN, R., PHILLIPS, D. & HART, I.R. (1986). Cell uptake, distribution and response to light of aluminium sulphonated phthalocyanine, a potential anti-tumour photosensitiser. *Br. J. Cancer*, **53**, 255.

COLERIDGE-SMITH, P.D., BOWN, S.G., MILLS, T.N., HOBBS, K.E.F. & SALMON, P.R. (1984). A quantitative study of photodynamic therapy in rats. *Proc. Brit. Med. Laser Assoc.*, 3rd annual conference London (*Abstract*).

DARWENT, J.R., McCUBBIN, I., PHILLIPS, D. (1982). Excited singlet and triplet state electron transfer reactions of Aluminium Sulphonated Phthalocyanine. *J. Chem. Soc. Faraday*, **2**, 78, 347.

EL-FAR, M.A. & PIMSTONE, N.R. (1985). The interaction of tumour-localising porphyrins with Collagen, elastin, gelatin, fibrin and fibrinogen. *Cell Biochem. Funct.*, **3**, 115.

FRIGERIO, N.A. (1962). *Metal phthalocyanines*. U.S. Patent No. 3,027,391 (Patented MAR 27 1962).

GOMER, C.J. & DOUGHERTY, T.J. (1979). Determination of [$^{3}$H] and [$^{14}$C] Hematoporphyrin derivative distribution in malignant and normal tissue. *Cancer Res.*, **39**, 146.

GREGORIE, H.B., HORGER, E.O., WARD, J.L. & 4 others (1968). Hematoporphyrin derivative fluorescence in malignant neoplasms. *Ann. Surg.*, **167**, 820.

HENDERSON, B.W., WALDOW, S.M. & DOUGHERTY, T.J. (1985). Interaction of photodynamic therapy (PDT) and hyperthermia. Tumour control and tumour cell survival after treatment *in vivo*. *Lasers Surg. Med.*, **5**, 139.

JORI, G., BELTRAMINI, M., REDDI, E., SALVATO, B. & 4 others (1984). Evidence for a major role of plasma lipoproteins as hematoporphyrin carriers *in vivo*. *Cancer Lett.*, **24**, 291.

JORI, G., REDDI, E., COZZANI, I. & TOMIO, L. (1986). Controlled targeting of different sub-cellular sites by porphyrins in tumour bearing mice. *Br. J. Cancer*, **53**, 615.

LIPSON, R.L., BALDES, E.J. & OLSEN, A.M. (1961). The use of a derivative of hematoporphyrin in tumour detection. *J. Natl Cancer Inst.*, **26**, 1.

MEW, D., WAT, CHI-KAT, TOWERS, G.H.N. & LEVY, J.G. (1983). Photoimmunotherapy: Treatment of animal tumours with tumour specific monoclonal antibody-hematoporphyrin conjugates. *J. Immunol.*, **3**, 1473.

McCUBBIN, I. (1985). Photochemistry of some water soluble phthalocyanines. *PhD Thesis, University of London.*

PIMSTONE, N.R., HORNER, I.J., SHAYLOR-BILLINGS, J. & GANDI, S.N. (1982). Haematoporphyrin augmented phototherapy: Dosimetric studies in experimental liver cancer in the rat. SPIE., 357. *Lasers Med. Surg.*, **60**.

PROFIO, A.E., DOIRON, D.R. (1981). Dosimetry considerations in phototherapy. *Med. Phys.*, **8**, 190.

ROUSSEAU, J., AUTENRIETH, D. & VAN LIER, J.E. (1983). Synthesis, tissue distribution and tumour uptake of [$^{99m}$Tc] Tetrasulfophthalocyanine. *Int. J. Appl. Radiat. isotopes*, **34**, 571.

ROUSSEAU, J., ALI, M., LAMOUREUX, G., LEBEL, E. & VAN LIER, J.E. (1985). Synthesis, tissue distribution and tumour uptake of $^{99m}$Tc and $^{67}$Ga-tetrasulfophthalocyanine. *Int. J. Appl. Radiat. Isotopes*, **36**, 709.

SPIKES, J.D. (1986). Phthalocyanines as photosensitisers in biological systems and for the photodynamic therapy of tumours. *Photochem. Photobiol.*, **43**, 691.

SVASSAND, L.O. & ELLINGSEN, R. (1983). Optical properties of human brain. *Photochem. Photobiol.*, **38**, 293.

SVASSAND, L.O. (1984). Thermal and optical dosimetry for photoradiation therapy of malignant tumours. In *Porphyrins in tumour Phototherapy*, Andreoni and Cubbedu (eds) p. 261. Plenum Press: New York.

TRALAU, C.J., BARTON, T., BARR, H., LEWIN, M.R. & BOWN, S.G. (1986). Uptake and distribution of Aluminium Sulphonated Phthalocyanine (AlSPc) in rats bearing dimethylhydrazine (DMH) induced colon tumours. *Proc. Brit. Med. Laser Assoc.*, 4th Annual Conference, London (*Abstract*).

VAN LIER, J.E., ALI, H. & ROUSSEAU, J. (1984). Phthalocyanines labeled with gamma-emitting radionucleotides as possible tumour scanning agents. In *Porphyrin Localisation and treatment of tumours*, Doiron, D.R. & Gomer, C.J. (eds) p. 315. Alan R. Liss: New York.

WEISHAUPT, K.R., GOMER, C.J. & DOUGHERTY, T.J. (1976). Identification of singlet oxygen as the cytotoxic agent in photoactivation of a murine tumour. *Cancer Res.*, **36**, 2326.


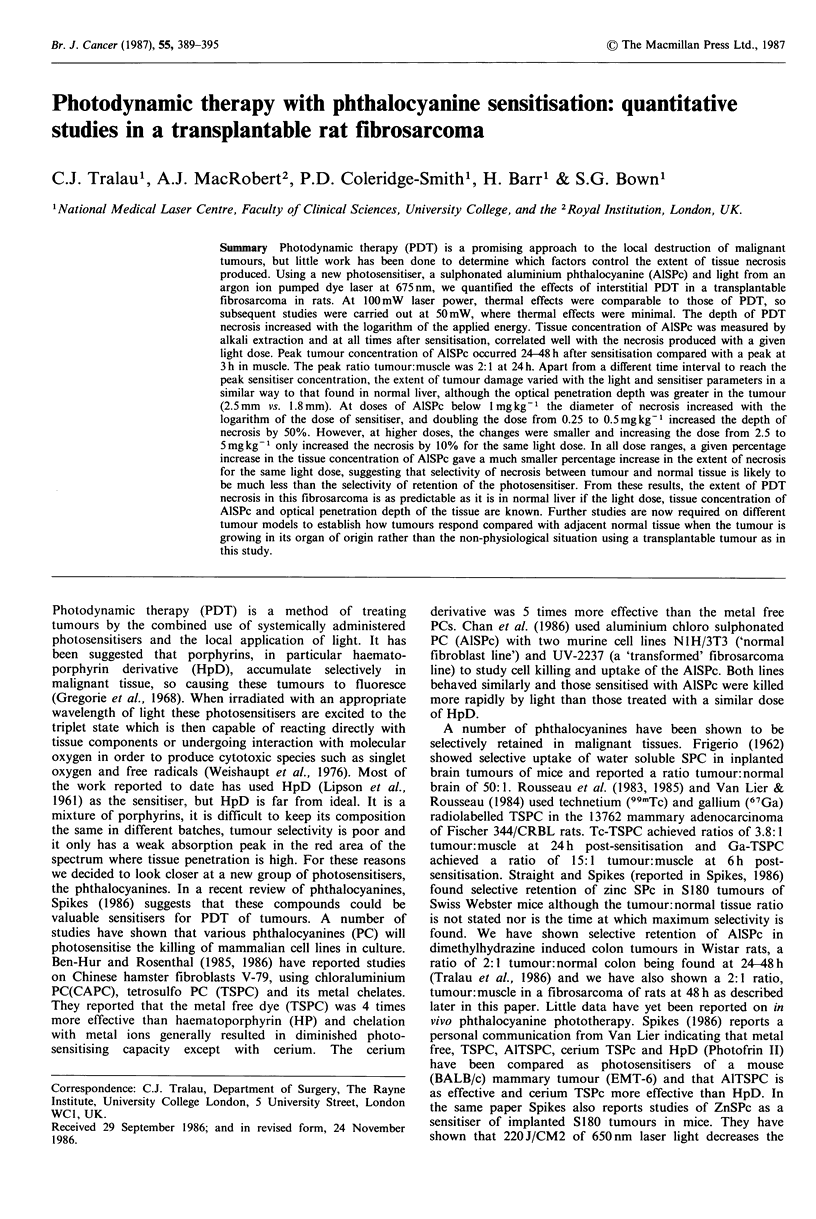

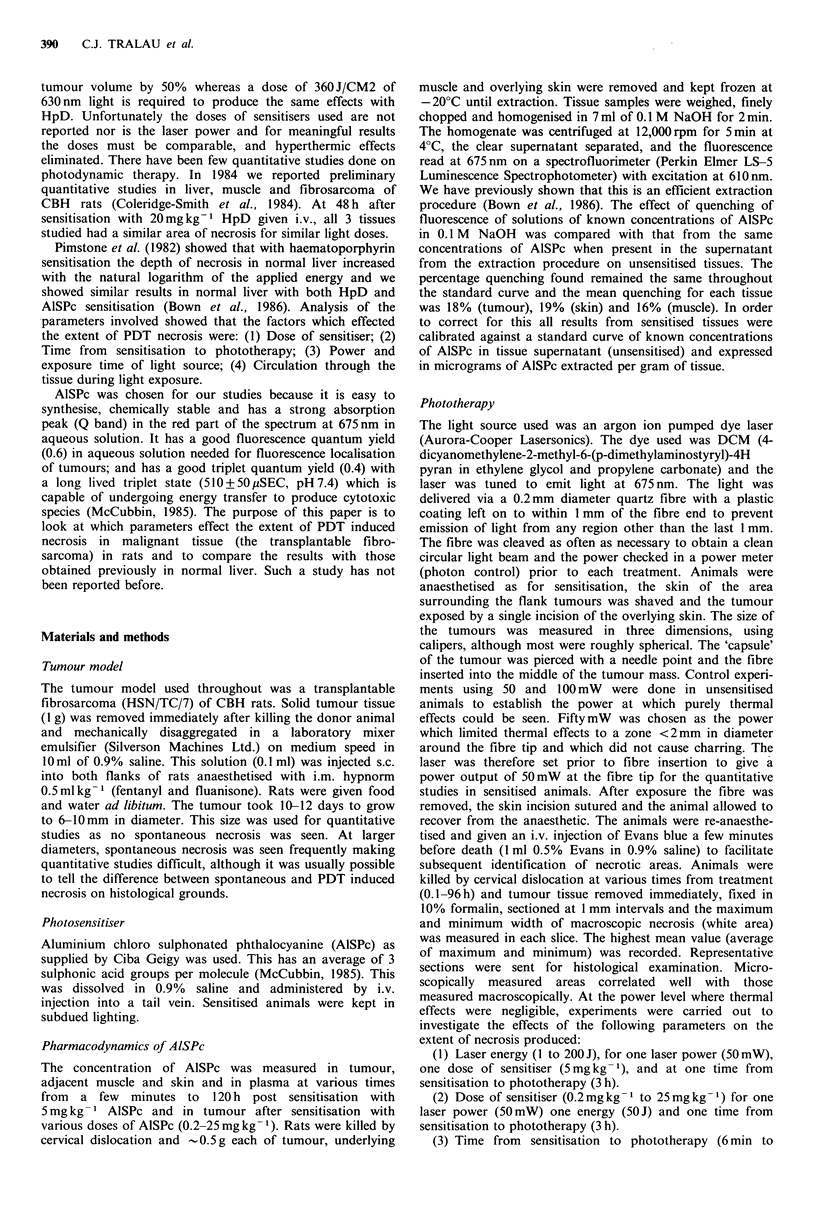

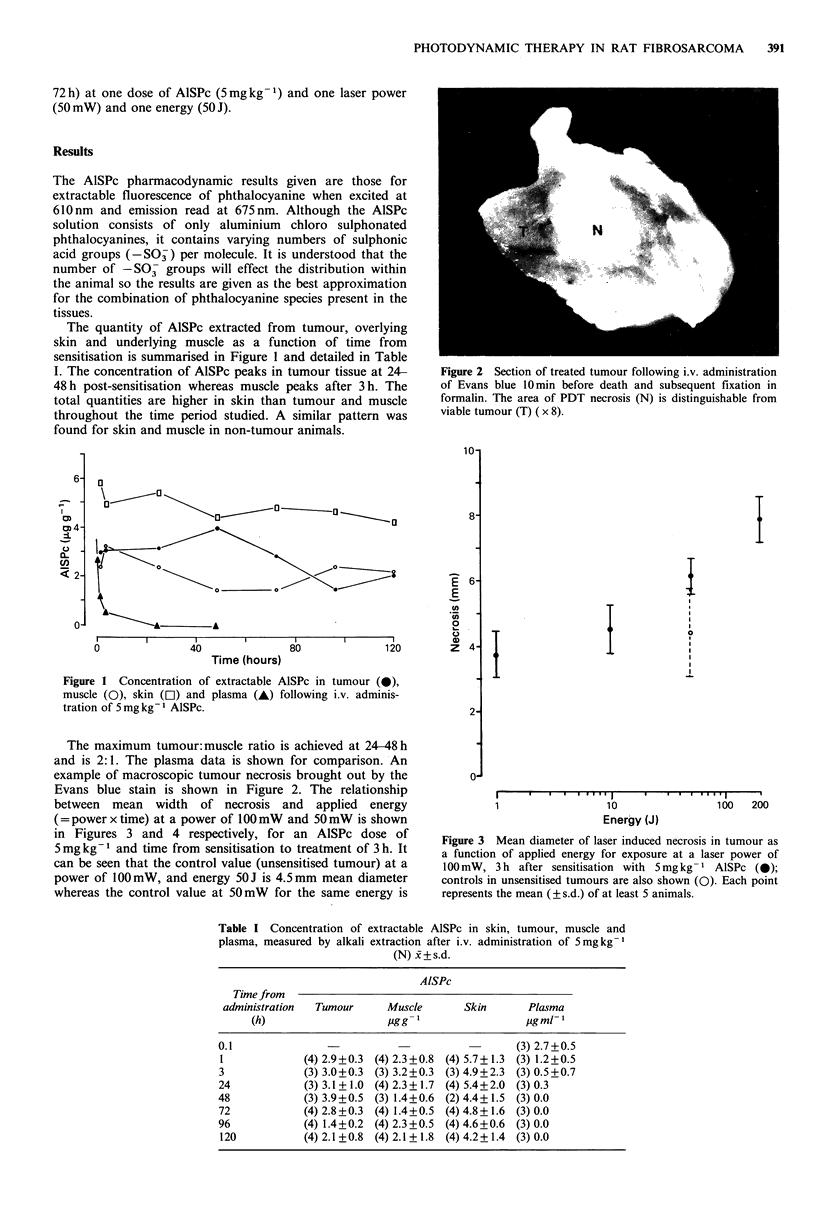

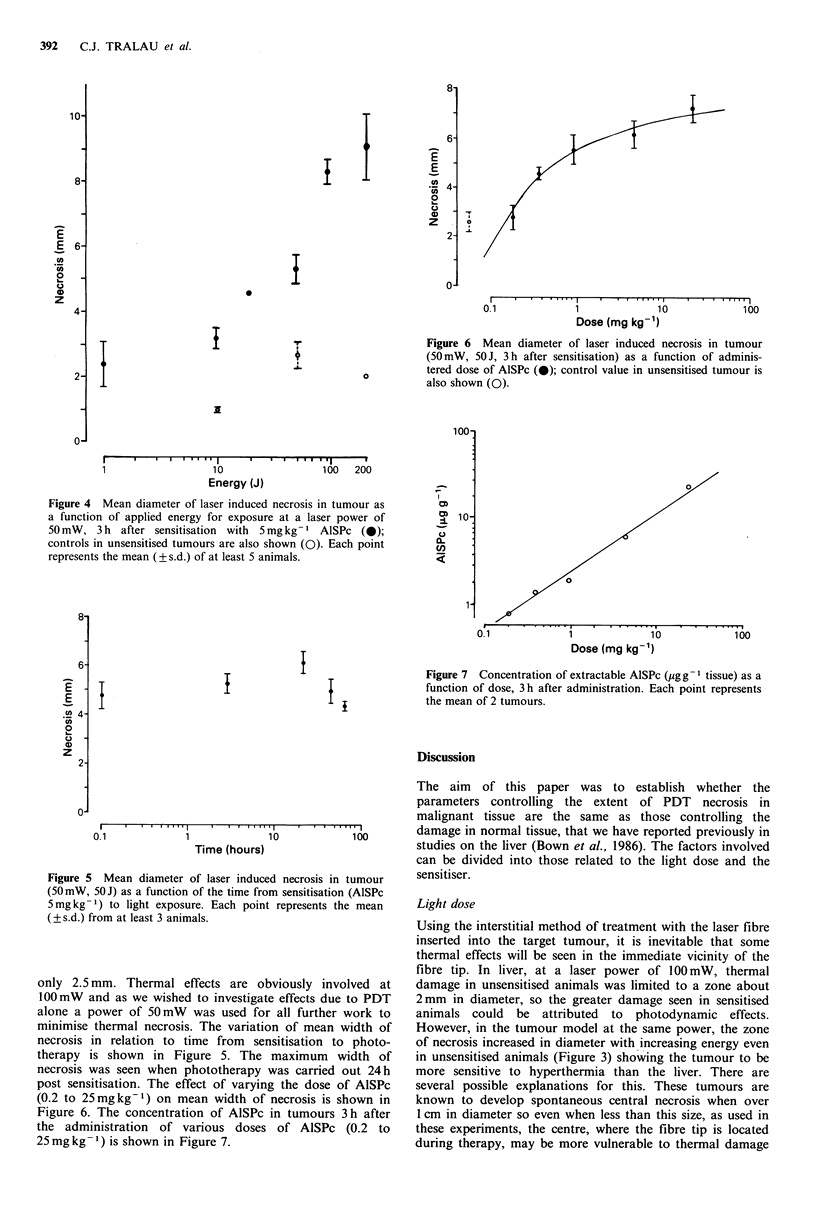

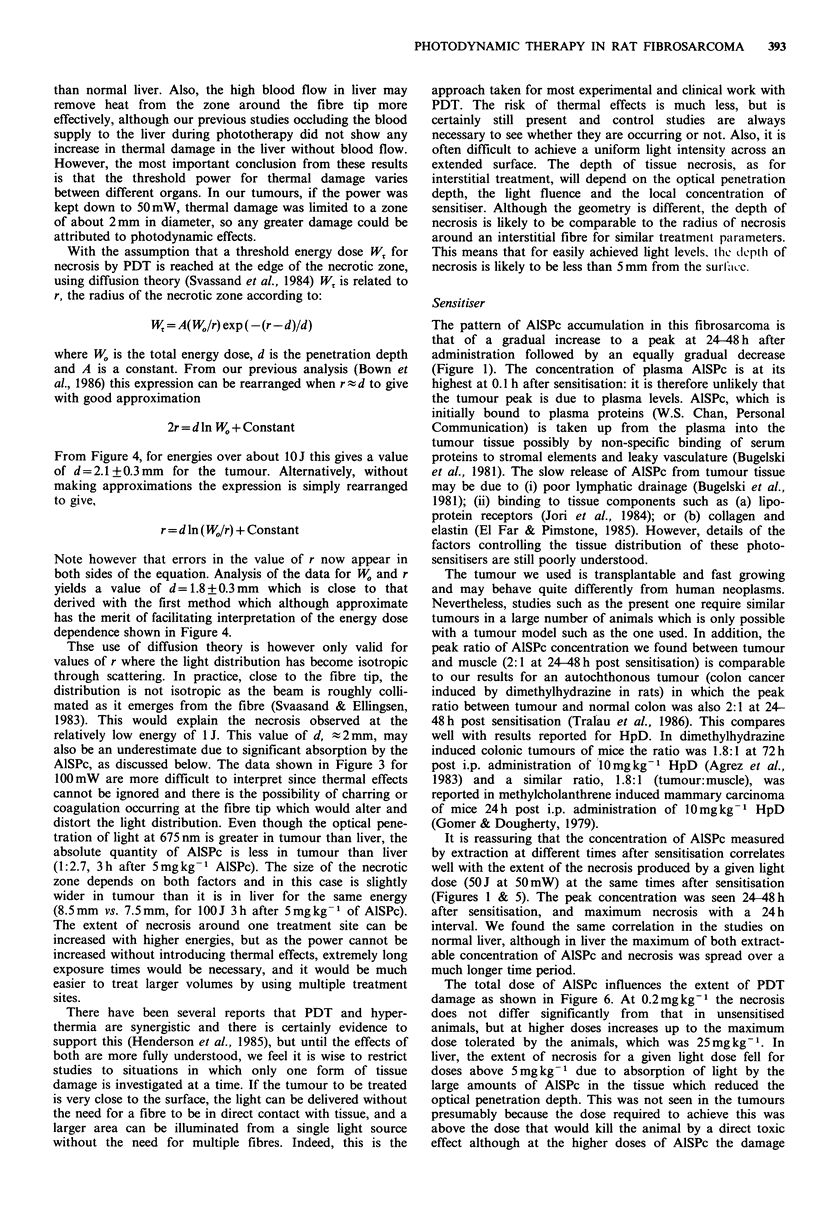

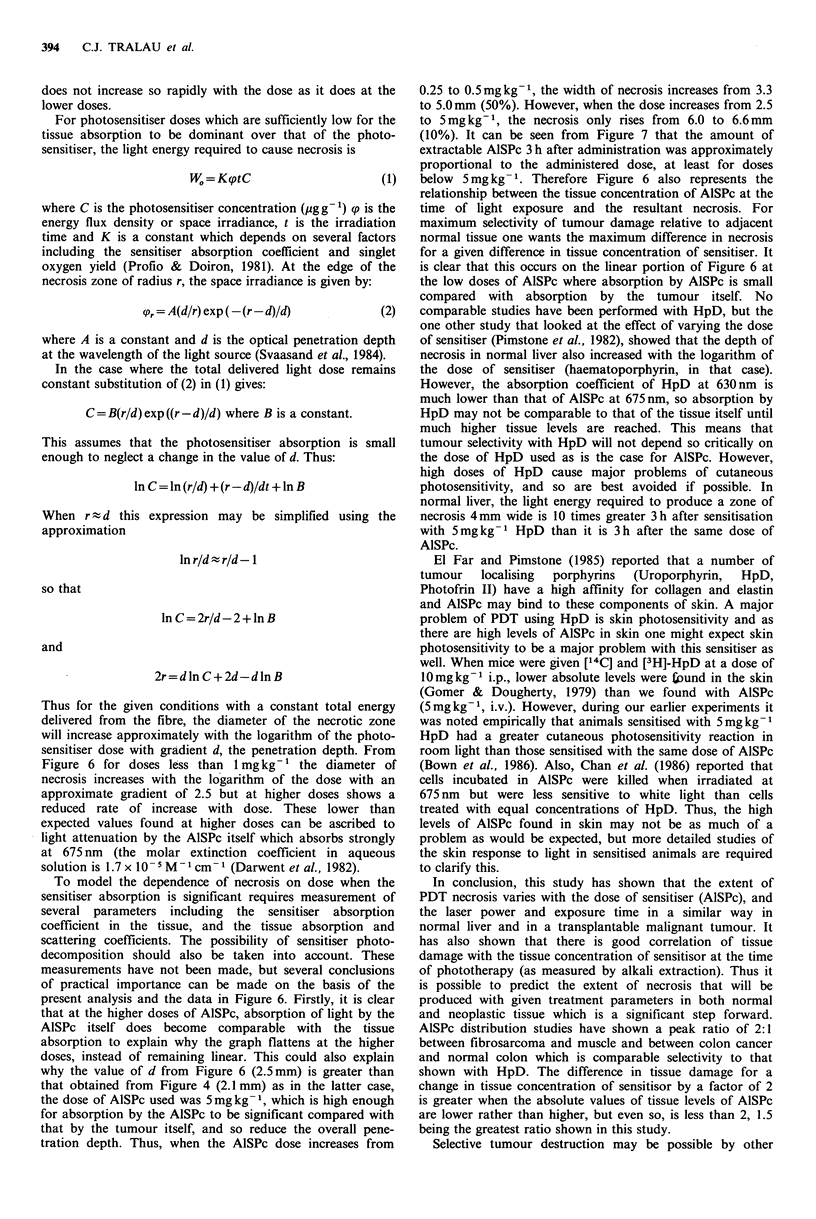

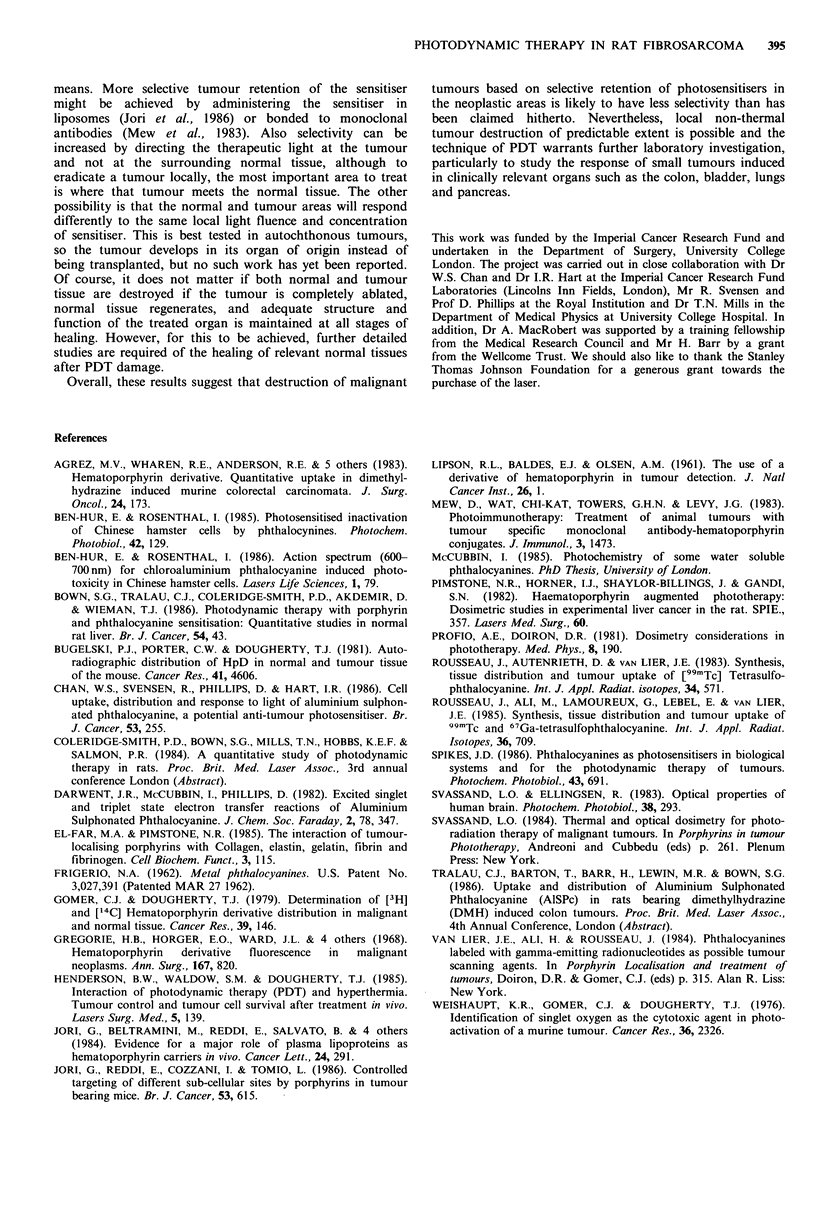


## References

[OCR_00971] Agrez M. V., Wharen R. E., Anderson R. E., Laws E. R., Ilstrup D. M., Cortese D. A., Shorter R. G., Lieber M. M. (1983). Hematoporphyrin derivative: quantitative uptake in dimethylhydrazine-induced murine colorectal carcinoma.. J Surg Oncol.

[OCR_00977] Ben-Hur E., Rosenthal I. (1985). Photosensitized inactivation of Chinese hamster cells by phthalocyanines.. Photochem Photobiol.

[OCR_00987] Bown S. G., Tralau C. J., Smith P. D., Akdemir D., Wieman T. J. (1986). Photodynamic therapy with porphyrin and phthalocyanine sensitisation: quantitative studies in normal rat liver.. Br J Cancer.

[OCR_00993] Bugelski P. J., Porter C. W., Dougherty T. J. (1981). Autoradiographic distribution of hematoporphyrin derivative in normal and tumor tissue of the mouse.. Cancer Res.

[OCR_00998] Chan W. S., Svensen R., Phillips D., Hart I. R. (1986). Cell uptake, distribution and response to aluminium chloro sulphonated phthalocyanine, a potential anti-tumour photosensitizer.. Br J Cancer.

[OCR_01024] Gomer C. J., Dougherty T. J. (1979). Determination of [3H]- and [14C]hematoporphyrin derivative distribution in malignant and normal tissue.. Cancer Res.

[OCR_01029] Gregorie H. B., Horger E. O., Ward J. L., Green J. F., Richards T., Robertson H. C., Stevenson T. B. (1968). Hematoporphyrin-derivative fluorescence in malignant neoplasms.. Ann Surg.

[OCR_01040] Jori G., Beltramini M., Reddi E., Salvato B., Pagnan A., Ziron L., Tomio L., Tsanov T. (1984). Evidence for a major role of plasma lipoproteins as hematoporphyrin carriers in vivo.. Cancer Lett.

[OCR_01045] Jori G., Reddi E., Cozzani I., Tomio L. (1986). Controlled targeting of different subcellular sites by porphyrins in tumour-bearing mice.. Br J Cancer.

[OCR_01050] LIPSON R. L., BALDES E. J., OLSEN A. M. (1961). The use of a derivative of hematoporhyrin in tumor detection.. J Natl Cancer Inst.

[OCR_01055] Mew D., Wat C. K., Towers G. H., Levy J. G. (1983). Photoimmunotherapy: treatment of animal tumors with tumor-specific monoclonal antibody-hematoporphyrin conjugates.. J Immunol.

[OCR_01071] Profio A. E., Doiron D. R. (1981). Dosimetry considerations in phototherapy.. Med Phys.

[OCR_01080] Rousseau J., Ali H., Lamoureux G., Lebel E., van Lier J. E. (1985). Synthesis, tissue distribution and tumor uptake of 99mTc- and 67Ga-tetrasulfophthalocyanine.. Int J Appl Radiat Isot.

[OCR_01075] Rousseau J., Autenrieth D., van Lier J. E. (1983). Synthesis, tissue distribution and tumor uptake of [99Tc]tetrasulfophthalocyanine.. Int J Appl Radiat Isot.

[OCR_01086] Spikes J. D. (1986). Phthalocyanines as photosensitizers in biological systems and for the photodynamic therapy of tumors.. Photochem Photobiol.

[OCR_01091] Svaasand L. O., Ellingsen R. (1983). Optical properties of human brain.. Photochem Photobiol.

[OCR_01115] Weishaupt K. R., Gomer C. J., Dougherty T. J. (1976). Identification of singlet oxygen as the cytotoxic agent in photoinactivation of a murine tumor.. Cancer Res.

[OCR_01015] el-Far M. A., Pimstone N. R. (1985). The interaction of tumour-localizing porphyrins with collagen, elastin, gelatin, fibrin and fibrinogen.. Cell Biochem Funct.

[OCR_01108] van Lier J. E., Ali H., Rousseau J. (1984). Phthalocyanines labeled with gamma-emitting radionuclides as possible tumor scanning agents.. Prog Clin Biol Res.

